# Unveiling Particulate Matter–Lung Interactions from Static Models to Dynamic Lung-on-Chips

**DOI:** 10.34133/research.1414

**Published:** 2026-09-02

**Authors:** Yixin Ren, Xi Deng, Mengqi Yang, Ziwei Liang, Yan Wei, Ting Liu, Weiwei Lan, Yiwei Shi, Di Huang

**Affiliations:** ^1^ Department of Biomedical Engineering, Research Center for Nano-biomaterials & Regenerative Medicine, Shanxi Key Laboratory of Functional Proteins, College of Artificial Intelligence, Taiyuan University of Technology, Taiyuan 030024, China.; ^2^ Institute of Biomedical Engineering, Shanxi Key Laboratory of Materials Strength & Structural Impact, Taiyuan University of Technology, Taiyuan 030024, China.; ^3^ NHC Key Laboratory of Pneumoconiosis, Shanxi Key Laboratory of Respiratory Diseases, Department of Pulmonary and Critical Care Medicine, The First Hospital of Shanxi Medical University, Taiyuan 030001, China.; ^4^ Henan Tuoren Medical Device Co., Ltd., Xinxiang 453431, China.

## Abstract

The pervasive inhalable particulate matter (PM) in the environment poses a marked threat to pulmonary health. However, the underlying pathogenic mechanisms remain poorly understood, largely due to the limitations of existing in vitro models in accurately mimicking the complex biological processes. This review critically examines the spectrum of in vitro models employed in PM research, spanning from traditional 2-dimensional models to advanced 3-dimensional (3D) constructs, including 3D bioprinted models and organoids, as well as the burgeoning field of lung-on-chips (LOCs). Each model type is meticulously assessed for its unique strengths and the challenges. A particular emphasis is placed on the innovative use of LOCs technology to replicate the dynamic nature of respiratory motion. The review delves into how integrating breath-mimicking stimuli can modulate the intricate interactions between PM and cellular/tissue interfaces. Ultimately, it unveils an innovative, highly integrated, and intelligent LOCs platform, offering a forward-looking insight into its evolution.

## Introduction

With the acceleration of industrialization and the rise in urbanization levels, air particulate pollution has become one of the core challenges in the global public health sector [1,2]. According to World Health Organization data, particulate matter (PM) exposure causes over 1 million premature deaths annually [3]. Among these pollutants, PM_2.5_ poses a particularly marked threat to lung health due to its high penetrating power and accumulation of toxic substances. The lungs serve as the primary target organ for PM entering the human body. Long-term exposure to PM can induce various respiratory diseases, including chronic obstructive pulmonary disease (COPD), pulmonary fibrosis, and lung cancer [4–7]. Although existing research has preliminarily revealed the association between PM and lung diseases, the specific mechanisms of action and the disease progression process require further clarification through subsequent studies [8,9].

As an organ with a complex hierarchical structure and composition, the different components of lungs are subjected to heterogeneous dynamic stresses during respiratory movements [10]. These unique and sophisticated physiological characteristics present substantial challenges in accurately reconstructing lung models in vitro. Previous studies have demonstrated that in vitro models not only facilitate the analysis of specific responses in human cells to PM exposure but also provide a powerful tool for systematically revealing the cellular and molecular toxicological mechanisms induced by PM [11]. Conventional in vitro cellular models, including 2-dimensional (2D) cell cultures, transwell systems, and 3-dimensional (3D) models, have been extensively employed in respiratory research; however, these models exhibit inherent limitations. Notably, they commonly rely on monocellular cultures, lack a physiological mechanical microenvironment that recapitulates the cyclic tensile strain of respiratory movement, and fail to reconstruct a functional air–blood barrier [12]. As a result, experimental conclusions drawn from such models, which poorly mimic the native in vivo structural and functional characteristics, are often unreliable, a critical factor contributing to the high attrition rate in pulmonary drug development [13,14]. Traditional animal models can systematically reflect the comprehensive response of organisms to PM exposure; they present substantial limitations that cannot be ignored, including pronounced species differences, high experimental costs, and ethical controversies [15–17].

Therefore, there is a clear need to develop a novel lung model that can faithfully simulate the human pulmonary microphysiological environment while also accommodating individual-specific variations. The rapid advancement of organoids and microfluidic organ-on-chips (OOCs) has made it possible to reconstruct the human lung microenvironment in vitro.

This review systematically elucidates the pulmonary toxicological mechanisms of inhalable PM in the environment. It evaluates various in vitro models applied to PM research, highlighting their advantages and challenges. The scope encompasses traditional 2D models, 3D models (3D bioprinting and organoids), and lung-on-chips (LOCs). We focus on exploring the innovative application of LOCs in dynamic respiratory motion and analyze the effects of breath stimulation integration on PM interacting with cellular as well as tissue interfaces. It offers new insights for health assessments of environmental pollutants alongside the prevention and treatment of respiratory diseases.

## PM-Induced Pulmonary Toxicology

### PM classification

PM refers to complex suspended matter composed of solid particles and liquid droplets in the air, typically formed through chemical reactions between pollutants in the atmosphere [18]. PM can be classified based on particle size, chemical composition, and emission sources [19]. In environmental health research, PM is most commonly categorized according to aerodynamic equivalent diameter (AED), including PM_10_ (AED ≤ 10 μm), PM_2.5_ (AED ≤ 2.5 μm), and PM_0.1_ (AED ≤ 0.1 μm), which are further grouped as coarse particles (PM_2.5–10_), fine particles (PM_0.1–2.5_), and ultrafine particles (PM_0.1_) [20] to reflect their distinct deposition patterns as well as biological effects. In the human respiratory system, the size variation of PM is a key factor influencing its penetration capacity. Consequently, different PM deposit in varying locations [21], thereby exerting diverse effects on human health [22–24]. Table 1 summarizes the deposition characteristics and effects of PM of different particle sizes on the human respiratory system.

**Table 1.** Deposition traits, human physiological impacts, and dominant molecular mechanisms of PM of various sizes

AED (μm)	Deposition characteristics	Effects on human physiology	Dominant molecular mechanisms
>10	Nasopharyngeal region; upper respiratory tract filtration	Sneezing, coughing, etc.	Mucosal irritation
PM_2.5–10_	Trachea and bronchi	Bronchial mucus hypersecretion; local inflammation	Airway inflammation; mucus hypersecretion through EGFR/MUC5AC signaling; activation of NF-κB and MAPK pathways
PM_0.1–2.5_	Penetrate into bronchioles and alveoli	Immune and inflammatory responses; cellular damage and barrier dysfunction; bronchiectasis, pulmonary edema, COPD	Oxidative stress; ROS-mediated inflammation; epithelial barrier disruption; NLRP3 activation; immune dysregulation
PM_0.1_	Alveolar compartment deposition; alveolar–capillary barrier penetration	Autophagy disorder; pulmonary barrier penetration; enter the systemic circulation to migrate to organs	Cellular internalization and organelle penetration; mitochondrial dysfunction; DNA damage response; epigenetic modifications; persistent transcriptomic reprogramming and senescence-related pathways

**Fig. 1.**
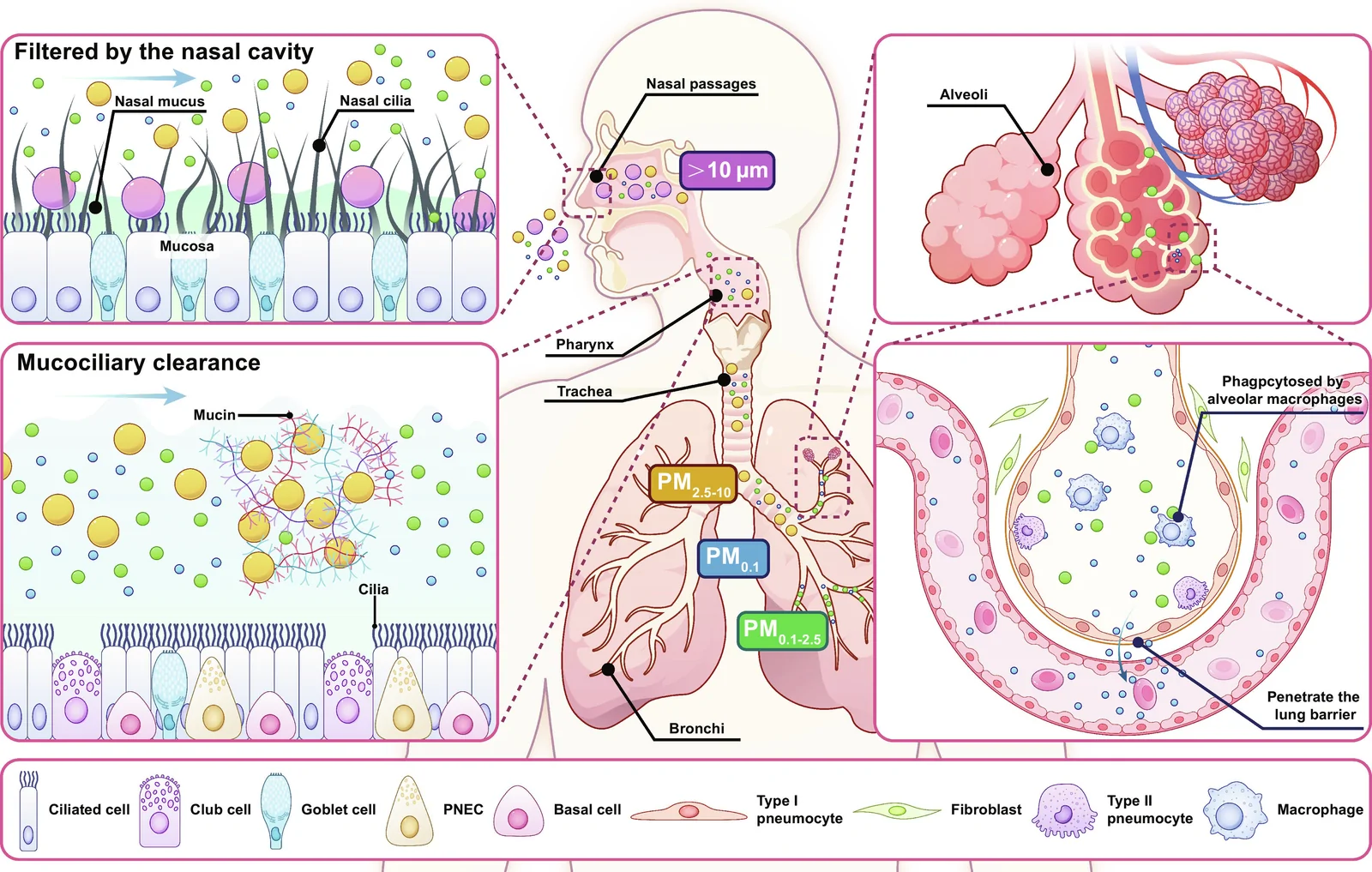
Author-generated schematic illustration of the deposition and clearance of PM with different AEDs in the human respiratory tract.

Specifically, PM with a diameter of more than 10 μm is primarily subject to inertial impaction. Unable to penetrate deep into the respiratory tract under air flow, most of it deposits in the nasal and pharyngeal regions. It is eliminated from the body through the physical interception of nasal hairs and nasal mucus, coupled with physiological reflexes such as sneezing and coughing [25]. Thus, it exerts a minimal direct impact on the lungs. In contrast, PM_2.5–10_ is mainly deposited in the tracheal and bronchial regions, trapped by airway mucins and slowly cleared via the mucociliary system. Prolonged exposure inhibits ciliary beating, induces mucus hypersecretion and airway epithelial inflammation, and is directly associated with rhinitis, pharyngitis, bronchitis, and other disorders [26]. Relying on Brownian diffusion and gravitational sedimentation, PM_0.1–2.5_ can penetrate deep into the bronchioles and alveoli [27], activating alveolar macrophages and epithelial cells to trigger oxidative stress and an inflammatory immune cascade. This leads to alveolar epithelial damage and barrier dysfunction, and is directly linked to the progression of bronchiectasis, pulmonary edema, and COPD [28]. Nearly all PM_0.1_ deposits in the alveolar region; it crosses the alveolar–capillary barrier via tight junction penetration and other pathways, and even enters the systemic circulation to migrate to organs such as the heart, brain, liver, and kidneys [29], causing severe health consequences, as illustrated in Fig. 1.

### PM-induced pulmonary toxicological mechanisms

PM that deposits in the lungs engages in complex interactions with various cell types, triggering a cascade of molecular and cellular responses, which ultimately leads to lung injury and disease [9]. The core toxicological mechanisms primarily involve multiple interconnected aspects such as oxidative stress, inflammatory responses, cytotoxicity, and genetic damage [1]. These combined mechanisms form the cornerstone of PM-induced pathological changes in the lungs.

Oxidative stress is regarded as one of the most fundamental initiating toxic mechanisms of PM, especially PM_2.5_ [30]. PM can act as a “carrier” to accumulate large amounts of metals and organic chemicals [31]. After pulmonary cells take up PM, reactive oxygen species (ROS) levels rise significantly. Excessive ROS overwhelm cellular antioxidant defense systems, leading to an imbalance in redox status. This not only directly causes irreversible damage to lipids, proteins, and DNA, but more importantly, ROS serve as key messengers capable of activating multiple critical intracellular signaling pathways. The nuclear factor κB (NF-κB) and mitogen-activated protein kinase (MAPK) pathways function as central hubs in PM-induced inflammatory responses [32], driving the transcription and expression of numerous pro-inflammatory cytokines and chemokines. Concurrently, PM and its components can also directly trigger NOD-like receptor pyrin domain-containing 3 (NLRP3) inflammasome activation [33], thereby promoting the secretion of pro-inflammatory cytokines such as interleukin-1β (IL-1β). These factors collectively recruit and activate additional immune cells to infiltrate the alveolar cavity and pulmonary interstitium, further amplifying the inflammatory cascade [34]. Thus, a vicious cycle of “oxidative stress–inflammation” is established, forming a crucial pathological basis for the onset and progression of chronic inflammatory diseases such as COPD and asthma. Notably, PM exposure induces pulmonary injury largely through epithelial–immune crosstalk, wherein epithelial cells release alarmins and cytokines that activate resident and recruited immune cells. This leads to macrophage polarization, neutrophil recruitment, and amplification of inflammatory signaling pathways such as NF-κB and NLRP3 inflammasome activation. Recent immune-enhanced organoids and LOCs incorporating macrophages or multi-immune cell compartments provide more physiologically relevant platforms to recapitulate these processes in vitro [35,36].

Persistent oxidative stress and intense inflammatory responses can cause direct damage to lung tissue structure, leading to cytotoxicity. Mitochondria are the primary targets of ROS attacks, and PM exposure can induce the release of pro-apoptotic factors from them [37]. Furthermore, activated NLRP3 inflammasomes not only promote the secretion of inflammatory cytokines but also elicit pyroptosis by cleaving the Gasdermin D protein, which further exacerbates the inflammatory microenvironment in the lungs [38]. PM can also influence gene expression by triggering epigenetic modifications [39]. Research indicates that PM exposure induces marked alterations in DNA methylation, with multiple genes involved in apoptosis and carcinogenesis [40]. Santoro et al. [41] demonstrated that lung epithelial cells, when singly exposed to real environmental concentrations of fine PM, may undergo epigenetic alterations in the expression of genes related to xenobiotic metabolism. Using both in vivo and in vitro models, Zeng et al. revealed that PM exposure increased METTL3-dependent m^6^A methylation of HDAC9 mRNA, thereby enhancing HDAC9 expression and sustaining MAPK activation through repression of DUSP9. This epigenetic regulatory axis promoted prolonged production of pro-inflammatory cytokines, providing evidence that RNA epigenetic modifications contribute to the chronic inflammatory responses induced by PM exposure [42]. Collectively, these findings suggest that PM exposure can induce long-lasting molecular alterations through multiple epigenetic mechanisms. This offers a new perspective for understanding the long-term health effects of PM. Additionally, although oxidative stress and inflammation are common responses across multiple PM size fractions, accumulating evidence suggests that particle size substantially influences the underlying molecular mechanisms [39,43]. Table 1 summarizes the dominant molecular mechanisms of PM of various sizes.

Meanwhile, the interaction between inhaled PM and the respiratory system is strongly influenced by extracellular surface barriers, particularly airway mucus and pulmonary surfactant. While mucus predominates in the conducting airways and serves as a primary defense mechanism through mucociliary clearance, pulmonary surfactant forms a thin lipid–protein film covering the alveolar surface and plays an essential role in regulating both respiratory mechanics and particle behavior [44]. Beyond its classical function of reducing surface tension and preventing alveolar collapse, surfactant directly interacts with inhaled particles, nanoparticles (NPs), and pathogens. These interactions can alter particle aggregation, diffusion, deposition patterns, and cellular uptake [45]. Importantly, surfactant proteins contribute to innate immune regulation by modulating macrophage activation, inflammatory signaling, and particle clearance [46]. Consequently, PM-induced responses observed in surfactant-deficient in vitro systems may differ substantially from those occurring in vivo. Notably, mucus is primarily modeled in conducting airway ALI systems, whereas surfactant production is typically evaluated in alveolar systems containing mature AT2 phenotypes and appropriate mechanical and biochemical cues. Since both mucus formation and surfactant functionality are highly dependent on cell source, differentiation status, and culture conditions, their presence and biological activity should be experimentally verified in each platform.

At the tissue level, the combined effects of the aforementioned mechanisms ultimately manifest as disruption of the alveolar–capillary barrier function. Excessive ROS generation and persistent inflammatory responses contribute to disruption of the alveolar–capillary barrier, resulting in increased permeability and loss of barrier integrity [47]. Beyond facilitating the translocation of PM and inflammatory mediators into systemic circulation, barrier injury may promote the leakage of plasma proteins and inflammatory exudates into the alveolar space. This process can lead to pulmonary edema, impaired gas exchange, and progressive respiratory dysfunction [48]. Importantly, the influx of serum-derived proteins and pro-inflammatory molecules may alter the composition and biophysical properties of pulmonary surfactant. Surfactant inactivation increases alveolar surface tension, compromises alveolar stability, and may contribute to alveolar collapse and further deterioration of respiratory function [44]. Consequently, PM-induced oxidative stress and inflammation can initiate a cascade of events linking barrier dysfunction, edema formation, surfactant impairment, and reduced pulmonary performance.

**Fig. 2.**
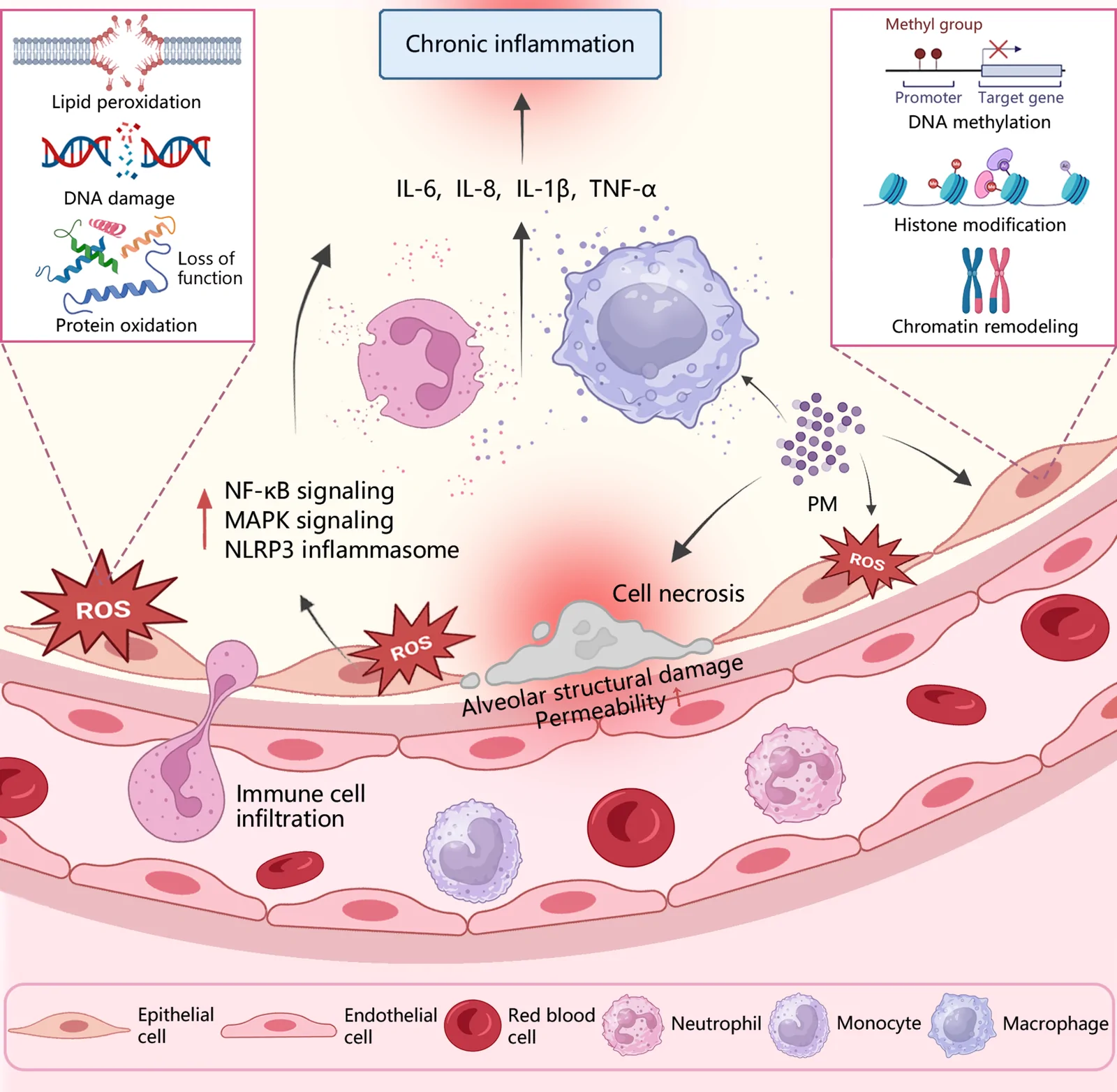
Author-generated schematic overview of the core mechanisms underlying PM-induced pulmonary toxicity. Created with BioRender.com.

A thorough understanding of the core mechanisms illustrated in Fig. 2 is crucial for accurately simulating and analyzing specific pathological processes using in vitro models, and it also provides a theoretical basis for developing targeted intervention strategies. It should be noted that PM-induced toxicity is not determined solely by particle size. While particle size strongly influences respiratory deposition patterns, tissue accessibility, and cellular uptake, the physicochemical properties of PM also play important roles in shaping biological responses [49].

Notably, the evidence supporting PM-induced oxidative stress, inflammation, barrier dysfunction, and cellular injury originates from multiple experimental systems, including in vitro cell cultures, animal models, and human studies. The magnitude and nature of these responses may vary substantially depending on the biological model employed. Consequently, interpretation of PM toxicological mechanisms should take into account the physiological relevance and inherent limitations of the underlying experimental platform.

## Pulmonary Effects of PM Using In Vitro Models

The construction of an in vitro lung model aims to replicate key microenvironmental features of the in vitro lung as closely as possible, thereby providing an experimental platform that more accurately reflects physiological conditions for PM-related research and other studies. The core objectives mainly encompass 2 aspects. First, to recapitulate the biological microenvironment by comprehensively considering pulmonary cellular composition, tissue architecture, and biochemical signaling. Second, to mimic the physical microenvironment, including the air–liquid interface (ALI), fluid shear stress, and cyclic breathing motions. Through the integrated simulation of these key elements, in vitro models are able to more realistically reflect the deposition patterns, clearance mechanisms, and toxicological effects of PM following its entry into the lung.

Notably, in the design of the in vitro model, it is crucial to strike a balance between model complexity and simulation fidelity. In general, enhancing the sophistication of in vitro models through the incorporation of a broader diversity of cell types, the engineering of more biomimetic 3D architectures, and the recapitulation of complex physiomechanical conditions can theoretically improve their ability to faithfully mimic the native in vivo tissue microenvironment. However, excessive complexity often introduces a range of challenges, including substantially increased technical difficulty, markedly higher costs, and more stringent experimental requirements, which may ultimately compromise the robustness, stability, and reproducibility of the model. Conversely, if the structure and conditions are overly simplified in pursuit of model convenience, although this may reduce experimental difficulty and improve operational efficiency, the absence of critical microenvironmental features will lead to marked discrepancies between the model and actual in vivo conditions. This may prevent the model from accurately reflecting the actual mechanism of PM action. Hence, in practical applications, appropriate in vitro models must be selected based on specific research objectives to achieve the optimal balance between research efficiency and result reliability.

**Fig. 3.**
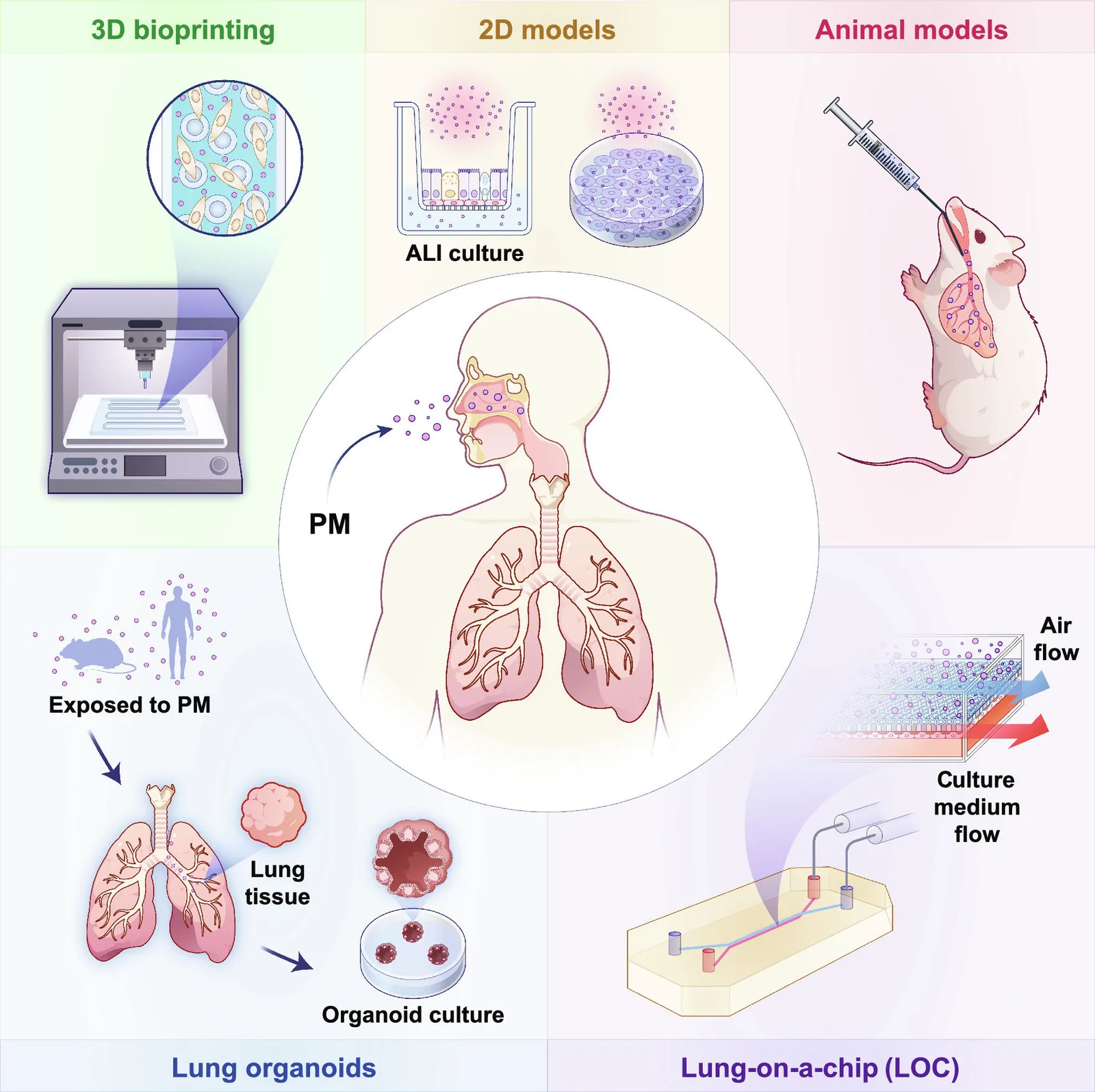
Author-generated schematic overview of models used to investigate PM-induced pulmonary effects.

A wide range of in vitro models has been developed for PM toxicology studies, and their value extends beyond simply providing alternative experimental platforms. Each model type is uniquely suited to address specific scientific questions, while simultaneously exhibiting distinct biological and technical limitations. Next, this review systematically introduces several commonly used models for PM research, including 2D models, 3D bioprinting, lung organoids, animal models, and LOCs, as illustrated in Fig. 3. Their respective advantages, challenges, and application characteristics are summarized in Table 2.

**Table 2.** Summary of pulmonary models for PM toxicity research

Models	Advantages	Challenges	Application features
2D models	Low cost; high throughput; easy operation	No cell–matrix interaction; no physical micro-environment simulation	Toxicological mechanism research; epithelial barrier function/PM trans-epithelial transport
3D bioprinting	Controllable 3D structures and multicellular arrangement; biomaterial-integrated mimicry	Complex and costly technology; bioink development; poor mechanical stimulation simulation	Realistic lung structure model development; PM effects investigation in 3D environments
Organoids	Self-organizing; multicellularity (spatial order and function preserved); robust self-renewal; disease modeling and personalized research suitable	Batch variations; no immune circulation networks; limited exogenous mechanical stimulation	PM effects on lung development and homeostasis; personalized risk assessment and drug screening
Animal models	Study of complete biological system responses, long-term effects, behavioral changes and complex pathology	Ethical concerns; species differences; high costs; long cycles; insufficient microscopic mechanistic study	Systemic physiological response study; in vitro finding validation
LOCs	Dynamic respiratory and blood flow shear force simulation; in situ monitoring and sensor integration; organ–organ interaction; personalized customization	Complex operation; no standardization; common material PDMS adsorption	Dynamic fluid and breathing motion effects on PM toxicity; authentic PM exposure and deposition

### 2D models

Studies on PM-induced pulmonary toxicity has historically been based on traditional 2D models, which are generally classified into conventional submerged culture systems and ALI culture configurations. Typically, PM toxicity experiments involve exposing cells to specific concentrations of PM with varying particle sizes and compositions under static conditions to investigate fundamental toxicological mechanisms. In addition, the transwell system allows the study of intercellular interactions and the construction of ALI cultures, providing a more physiologically relevant environment for pulmonary cells. Importantly, ALI should be considered a culture configuration rather than an independent model type. Depending on experimental design, ALI can be incorporated into different in vitro models to improve physiological relevance for inhalation exposure studies.

The cells constituting the lung are diverse. Reports in the literature regarding in vitro PM exposure studies for lung toxicity using human-derived cells are typically based on cancer cell lines such as A549 and Calu-3, immortalized cell lines like BEAS-2B and huAEC, as well as primary cells including AT1 and AT2 [50]. In 2020, Liu et al. first assessed the adverse effects of silicon nanoparticles (SiNPs) exposure on pulmonary epithelial tight junctions and investigated potential molecular mechanisms through ROS/extracellular signal-regulated kinase (ERK) pathway and protein degradation [51]. Results indicated that SiNPs significantly decreased tight junction proteins ZO-1, ZO-2, and occludin in BEAS-2B, increased ROS production, and activated the MAPK signaling pathway. Moreover, the ALI culture configuration established using the transwell system serves as a crucial tool for investigating barrier function and PM in epithelial transport. At the same time, alveolar macrophages play a vital role in immune responses [52], while the human monocytic leukemia cell line 1 (THP-1) is commonly used as a substitute for primary human macrophages and is widely employed to study immune-related mechanisms following PM exposure. To better analyze the intricate interactions among different cell types following PM_2.5_ exposure, Wang et al. [53] established single-cell, dual-cell, and triple-cell coculture models using the transwell system, comprising A549 epithelial cells, THP-1 macrophages, and EA.hy926 endothelial cells. The results showed that particles could pass through the epithelial barrier into the endothelium, and PM_2.5_ caused inflammatory response in A549 epithelial cells and EA.hy926 endothelial dysfunction. Importantly, PM_2.5_-induced biological adverse reactions were more pronounced in the triple-cell coculture system compared to the dual-cell coculture system. This demonstrates that communication between different lung cell types influences epithelial barrier properties and modulates immune-inflammatory responses to exogenous stimuli. Accordingly, coculture systems should be considered over static monocultures, particularly when investigating mechanisms involving cell–cell communication, immune regulation, or particle translocation studies [54,55]. Meanwhile, although immortalized and cancer-derived cell lines have played a pivotal role in advancing our understanding of PM-induced pulmonary toxicity, their physiological relevance warrants careful consideration. For example, although A549 cells are frequently used as a surrogate for AT2 cells, they only partially reproduce the phenotype and functional properties of native AT2 cells. In particular, their capacity to synthesize, secrete, and regulate pulmonary surfactant differs substantially from that observed in vivo [56]. Likewise, transformed and immortalized cell lines may exhibit altered inflammatory signaling, metabolic activity, proliferation capacity, and stress responses compared with primary epithelial cells, which should be considered when interpreting PM-induced toxicological outcomes [57]. To improve translational relevance, increasing attention has been directed toward primary human airway and alveolar epithelial cells, which more closely preserve native cellular phenotypes and functional characteristics. In addition, stem cell-derived epithelial models provide a promising alternative by enabling the generation of physiologically relevant and patient-specific cell populations. Table 3 compares different lung cell types in PM toxicology studies.

**Table 3.** Comparison of different lung cell types for PM toxicology studies

Lung cell types	Advantages	Limitations	Recommended application
Cancer cell lines	Robust, reproducible, high throughput	Cancer-derived, altered signaling	Initial mechanistic screening
Immortalized cell lines	Easy culture; reproducible	Immortalization-related changes	Oxidative stress, inflammation studies
Primary cells	High physiological relevance	Donor variability, limited lifespan	Validation of key findings
Stem cell-derived models	Patient-specific, renewable	Cost, differentiation variability	Precision toxicology

Traditional 2D cell culture models are primarily designed to investigate direct cellular responses upon PM exposure, including elucidating oxidative stress pathways, inflammatory signaling cascades, DNA damage, mitochondrial dysfunction, and cell death mechanisms. Typical measurements include intracellular ROS generation, mitochondrial membrane potential, DNA damage markers, cell viability, and apoptosis. Among 2D models, coculture and ALI systems are particularly useful for studying barrier disruption, inflammatory crosstalk, particle translocation, and immune recruitment after PM exposure. Transepithelial electrical resistance (TEER), permeability assays, tight junction protein expression, and particle translocation assays are widely used to assess epithelial integrity. Meanwhile, inflammatory cytokines and chemokines are routinely quantified to evaluate intercellular crosstalk and immune activation. The simplicity, low cost, and high reproducibility of 2D models support mechanistic research and high-throughput screening for PM toxicity. However, a major limitation of conventional submerged cultures is the absence of physiologically relevant mucus and pulmonary surfactant layers, which can substantially alter PM transport and cellular responses. Additionally, the lack of 3D structural organization and dynamic biomechanical environment present in native lungs limits their physiological relevance and often results in poor prediction of tissue-level responses observed in vivo.

### 3D bioprinting

3D bioprinting enables the precise layering of cell-laden bioinks according to predesigned 3D digital models to construct in vitro tissue models with complex anatomical structures and high cellular activity [58]. Compared to traditional 2D models, it offers unique advantages in simulating the fine structures and functions of the human lung [59] and holds great potential as a powerful tool for studying PM deposition, toxicological mechanisms, and disease progression.

**Fig. 4.**
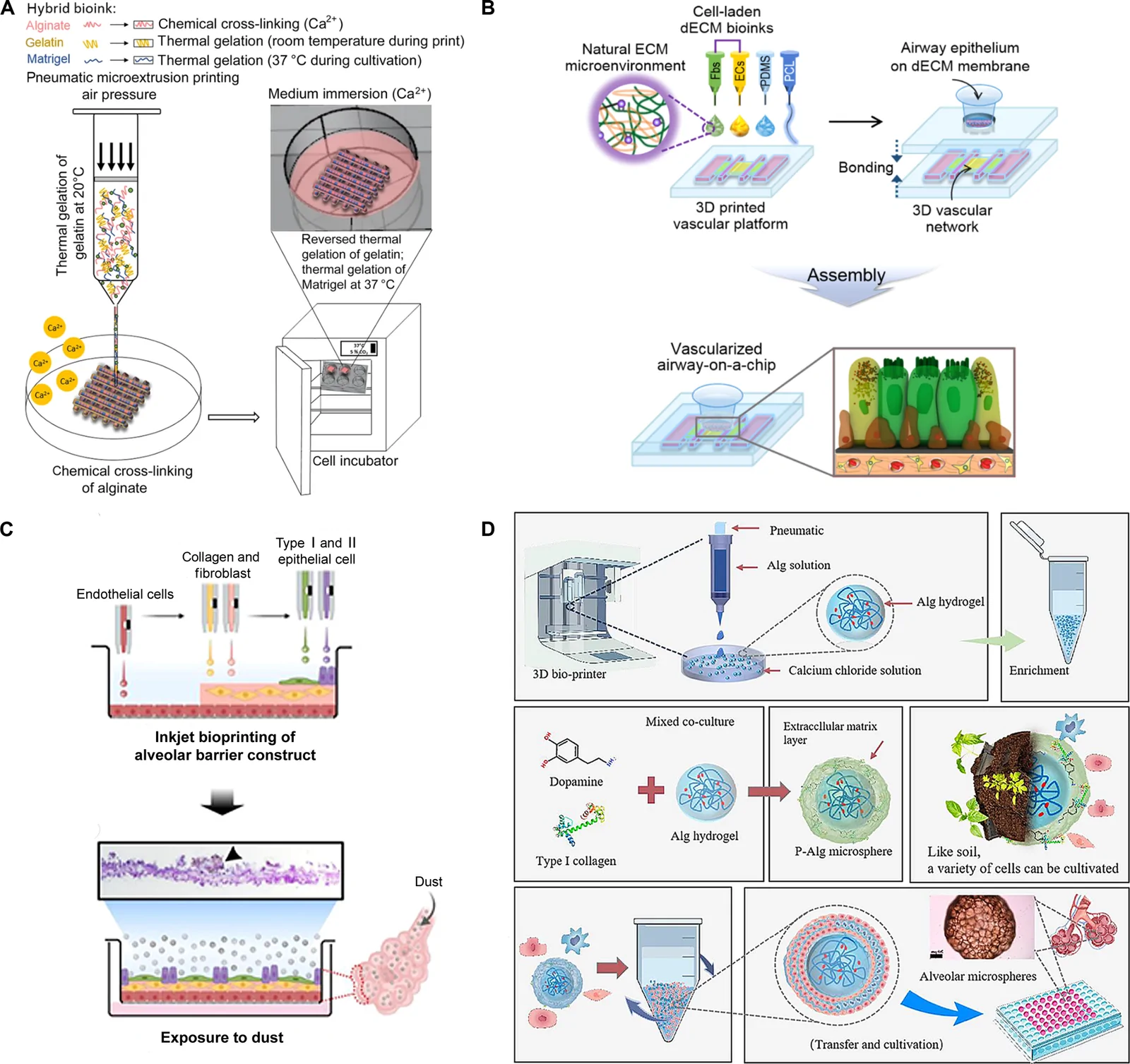
3D bioprinting technologies in pulmonary physiological and pathological model constructions. (A) After mixing the bioink with cells and preliminary cross-linking with Ca^2+^, the hydrogel was formed via pneumatic extrusion; gelatin thermogelation maintained the printed structure, and alginate achieved complete cross-linking in CaCl_2_ solution. During incubation at 37 °C, gelatin dissolved, and Matrigel gelation took over structural support. Adapted from Berg et al. [60]. (B) Based on the natural extracellular matrix (ECM) microenvironment, a vascular platform was fabricated via 3D bioprinting of cell-laden decellularized extracellular matrix bioinks and then assembled with a transwell airway epithelium model to form the vascularized airway-on-a-chip. Adapted from Park et al. [61]. (C) An alveolar barrier was constructed by sequentially stacking endothelial cells, fibroblast-laden collagen, and type I/II alveolar epithelial cells via inkjet bioprinting, followed by subsequent exposure to dust particles. Adapted from Kang et al. [62]. (D) Alginate hydrogel microspheres were fabricated via 3D bioprinting, surface-modified with dopamine and type I collagen to generate P-Alg microspheres, and cocultured with various cells to construct alveolar microspheres. Adapted from Wan et al. [63].

A research team created a 3D bioprinted lung model by optimizing bioink composition (Fig. 4A) [60]. Although this platform was established for influenza A virus infection studies, it demonstrated the ability of bioprinted lung constructs to sustain long-term culture and reproduce key pulmonary biological responses, including epithelial inflammatory reactions following external stimuli. Such features are highly relevant to PM toxicology, as they provide a more biomimetic microenvironment for investigating PM-induced cellular injury and inflammatory responses than conventional 2D culture systems. Furthermore, Park et al. [61] utilized 3D cell printing to develop a functional airway chip model that reproduces functional tissue microarchitecture and a 3D natural vascular network (Fig. 4B). In this study, ALI culture was used to induce the maturation and differentiation of airway epithelium. Additionally, the model successfully reconstituted the inflammatory response elicited by pathophysiological stimuli and analyzed the impact of functional vasculature on the inflammatory response of the airway epithelium. In 2022, Kang et al. created an alveolar barrier model using inkjet 3D printing to evaluate the effects of fine airborne particles, such as dust, on lung tissue (Fig. 4C) [62]. This model simulated the 3D structure of the alveolar barrier and investigated the impact of different concentrations of PM on cytotoxicity, alveolar barrier stiffness, and pulmonary surfactant secretion. It contributed to understanding the mechanisms by which PM caused pulmonary pathology. A recently published study constructed artificial lung tissue structure with 3D-inkjet bioprinting for modeling, research, and applications in acute lung injury diseases (Fig. 4D) [63]. Although the study primarily focused on the mechanisms of lung injury, this platform can utilize different cell types to build various 3D artificial lung tissues, providing a highly adjustable and reproducibly constructed potential model for subsequent PM exposure experiments.

3D bioprinted lung constructs provide a more biomimetic microenvironment by enabling spatial control over cell distribution and ECM composition. These models are particularly advantageous for studying how tissue architecture influences PM deposition, penetration, and local cellular responses. Furthermore, the ability to fabricate customized structures offers opportunities to investigate region-specific toxicity within the respiratory tract. In 3D bioprinting models, tissue-level assessments become increasingly important. Relevant readouts include cell viability throughout the construct, spatial distribution of PM, ECM remodeling, inflammatory mediator production, and alterations in tissue architecture. These measurements help evaluate how structural complexity influences PM toxicity. Nevertheless, current bioprinted tissues still face challenges regarding printing resolution, bioink compatibility, long-term viability, and the recreation of functional vascular and immune components [64,65]. These limitations may affect their ability to fully recapitulate chronic PM-induced pathological processes.

### Lung organoids

Organoids are 3D micro-organ structures formed by inducing stem cells to differentiate in vitro. They possess self-organizing capabilities and partial organ functions, enabling the simulation of organ development processes and disease pathological features [66]. This provides a new model with higher physiological relevance for studying the actual biological effects of PM within the human body [67].

**Fig. 5.**
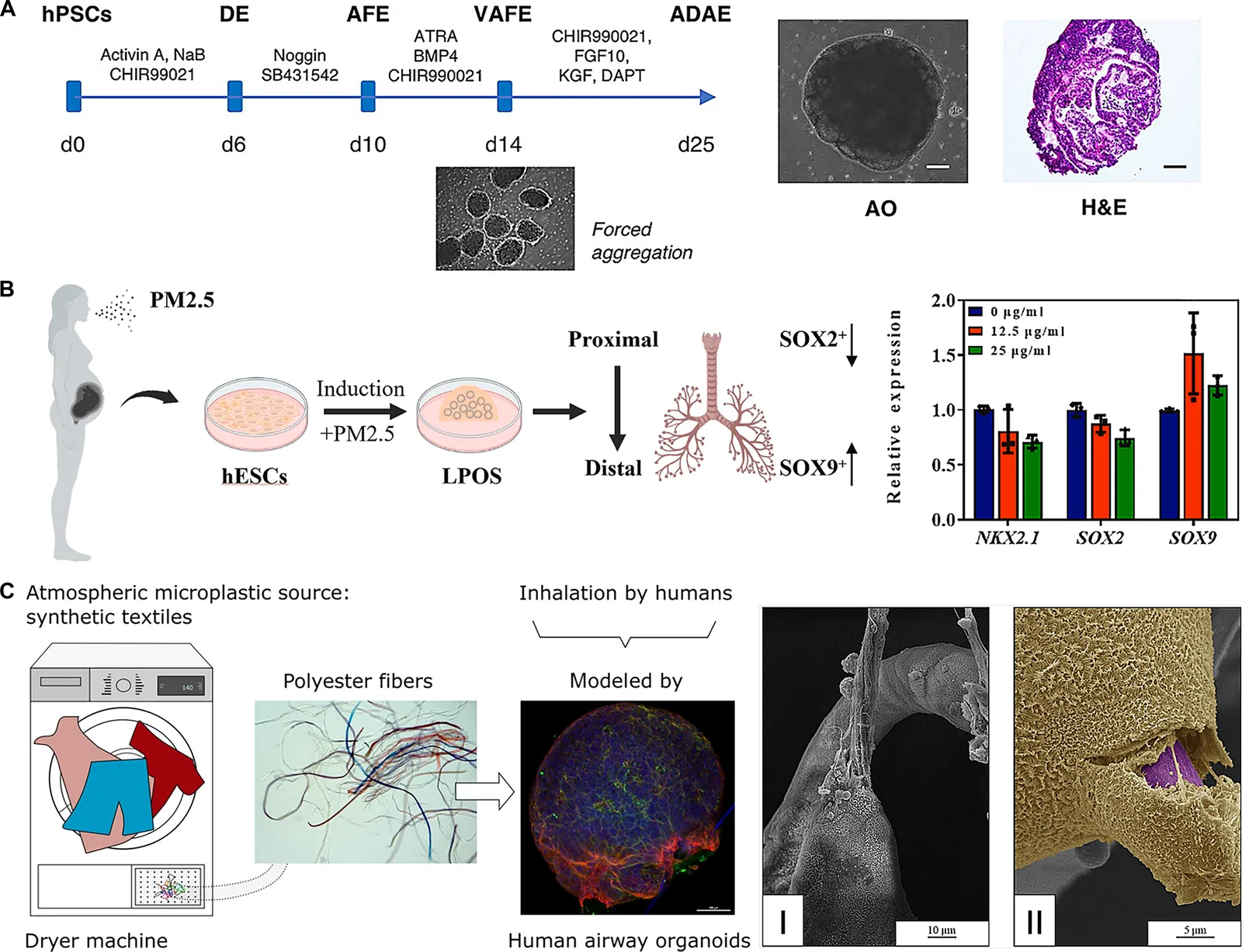
Lung organoid models to evaluate PM and fiber pulmonary impacts. (A) An hPSC-derived AO model was established for dPM_2.5_ developmental toxicity assessment; uniform hPSCs aggregates were induced to mature into AOs with alveolar sac-like structures and epithelial layers via optimized protocols. Scale bars: 100 μm. Adapted from Kim et al. [68]. (B) H9 hESC line was used to generate LPOs mimicking fetal lung early development; PM_2.5_ exposure during LPOs induction altered the expression of lung progenitor cell markers NKX2.1, SOX2, and SOX9, which regulate proximal–distal airway specification. Adapted from Wang et al. [69]. (C) MPF exposure did not affect airway organoid growth but induced polarized cell growth along fibers and fiber encapsulation by organoids, with implications for long-term lung epithelial repair. Adapted from Winkler et al. [70].

In 2020, Kim et al. [68] employed a system of alveolar organoids (AOs) differentiated from human pluripotent stem cells (hPSCs) to systematically evaluate the impact of diesel particulate matter (dPM_2.5_) on alveolar tissue development and cellular status (Fig. 5A). Research found that dPM_2.5_ treatment disrupted the differentiation process of alveolar epithelial cells, up-regulated genes associated with oxidative stress and inflammation, promoted epithelial–mesenchymal transition, activated the ERK signaling pathway, and might increase susceptibility to viral infections. This study validates lung organoids as a viable in vitro model for environmental toxicology. Additionally, another group examined the exposure effects of PM_2.5_ at different stages of early lung development using lung bud tip organoids (LPOs) derived from human embryonic stem cells (hESCs) (Fig. 5B) [69]. The study revealed that PM_2.5_ significantly impaired the cell proliferation of LPOs and disrupted the expression of several key transcription factors during early fetal lung development, further underscoring the substantial health risks of prenatal PM_2.5_ exposure to respiratory system development. Among air pollutants, the majority of microplastics (MP) consist of microplastic fibers (MPFs) from various sources. Winkler et al. proposed applying innovative human organoid models for testing human exposure to microplastics/nano-plastics and other particulate pollutants. They constructed human airway organoids from adult stem cells and discovered that nondegradable fibers present during the repair phase of damaged lung epithelium might lead to their encapsulation by the repairing tissue [70]. However, the long-term effects remain unclear. Moreover, the expression of the *SCGB1A1* gene was significantly reduced, a gene frequently used as a biomarker for monitoring lung injury (Fig. 5C). Notably, airway and lung organoids used for PM research are not biologically equivalent systems. Their responses to PM exposure may vary considerably depending on cellular origin, differentiation state, and anatomical identity. Consequently, the selection of organoid models should be guided by the specific biological question being addressed [71]. Patient-derived proximal airway organoids are particularly useful for investigating acute epithelial responses and interindividual variability, whereas AOs and iPSC-derived lung organoids are more appropriate for studying PM-induced alveolar damage, fibrosis, and regenerative processes.

Among in vitro models studying the influence of PM on pulmonary diseases, lung organoids and 3D bioprinted lung constructs represent more advanced 3D model systems than 2D cell cultures, each possessing distinct advantages and limitations. Compared to 3D bioprinting models that rely solely on manual arrangement of cells and matrix, organoids better reflect the tissue complexity and functional networks characteristic of organ hierarchies. PM-induced pathologies, including heterogeneous responses across cell populations, dysregulated cell communication, and impaired tissue barrier integrity, are often difficult to faithfully recapitulate using only predefined bioprinted constructs [72]. Consequently, lung organoids are well suited for investigating epithelial differentiation, stem cell responses, tissue regeneration, and chronic remodeling following PM exposure. Patient-derived organoids additionally provide a promising platform for studying interindividual variability and susceptibility to environmental pollutants. For these models, functional analyses often focus on epithelial differentiation, stem cell activity, regenerative capacity, and pathological remodeling. Meanwhile, AOs containing differentiated AT2 cells may partially reproduce endogenous surfactant production, providing advantages over conventional monocultures when investigating particle-surfactant interactions. However, most organoid systems lack physiological airflow, vascular perfusion, immune surveillance, and mechanical stimulation [73]. The enclosed 3D structure may also create diffusion gradients that influence particle exposure and distribution [74].

### Animal models as reference systems for evaluating advanced in vitro platforms

Although this review primarily focuses on in vitro platforms, a brief overview of animal models is provided because they have historically contributed substantially to the understanding of PM-induced pulmonary effects and have served as important references for evaluating toxicological responses at the organism level. The major advantage of animal models lies in their preservation of the entire immune system, blood circulation, and complex pulmonary tissue microenvironment. This makes them well suited for investigating the multilevel effects of PM exposure, including systemic inflammation, intercellular crosstalk, and alterations in respiratory function, all of which are challenging to fully recapitulate in conventional in vitro models.

Within the field of PM research, by establishing respiratory exposure or intratracheal administration models in rodents such as mice and rats [75], researchers can observe the impact of PM on lung structure and function at the whole-organ level [76,77]. Animal models also can provide direct evidence for the mechanisms of PM-induced pulmonary toxicology. To investigate the effects of PM_2.5_ on airway smooth muscle and its underlying mechanisms, Cheng et al. employed an inhalation exposure model in mice. Their findings revealed that inhaled PM distributed within airway smooth muscle bundles, accompanied by increased airway smooth muscle bundles and enhanced collagen deposition. Subsequent in vitro experiments confirmed that PM_2.5_ induced the senescence-associated secretory phenotype in airway smooth muscle cells, which served as a key factor promoting airway remodeling [78]. Another study team found that exposure to PM_10_ and PM_2.5_ exacerbated lung inflammation, fibrosis, oxidative stress, and apoptosis in mice, thereby inducing lung injury. The adverse effects induced by PM_2.5_ were more pronounced than those caused by PM_10_ [79].

Although animal models have made important contributions, their inherent limitations cannot be overlooked [15,16]. These primarily include the following: species differences are a key factor affecting the extrapolation of model results to humans [80,81]; ethical concerns are receiving increasing attention alongside the promotion of the “3R” principle [82]; and bottlenecks such as low throughput, high costs, and lengthy timelines. In summary, animal models can serve as valuable historical references and occasional contextual tools, but are not direct surrogates for human responses. With the rapid advancement of human-relevant in vitro platforms, combined with standardized exposure and dosimetry strategies, these next-generation models are increasingly enabling more precise mechanistic investigations of PM-induced pulmonary toxicity and may complement or reduce reliance on conventional animal approaches.

### Lung-on-chips

The OOCs represent an emerging class of in vitro models that integrate key functional elements of native organ microenvironments within a microfluidic chip [83,84]. This approach aims to mimic the structure and function of human organs, thereby reproducing physiological and pathological processes in an in vitro setting. Traditional in vitro models struggle to apply physical stimuli such as fluid shear forces and cyclic tensile stresses to simulate the true mechanical microenvironment of lung tissue during respiration, whereas LOCs can address this issue. Overall, the LOCs support coculture of multiple cell types (e.g., epithelial cells, endothelial cells, and immune cells) and can simulate breathing movements through cyclic mechanical stretching. Many LOCs also incorporate ALI conditions on the epithelial side while maintaining continuous perfusion in the vascular channel. This configuration more closely mimics the in vivo respiratory microenvironment and enables direct aerosol deposition onto epithelial surfaces, facilitating realistic assessment of PM-induced barrier dysfunction, inflammation, and tissue injury. In summary, LOCs reproduce the physiological functions and mechanical microenvironment of the lung, providing an advanced platform for PM lung toxicity research.

**Fig. 6.**
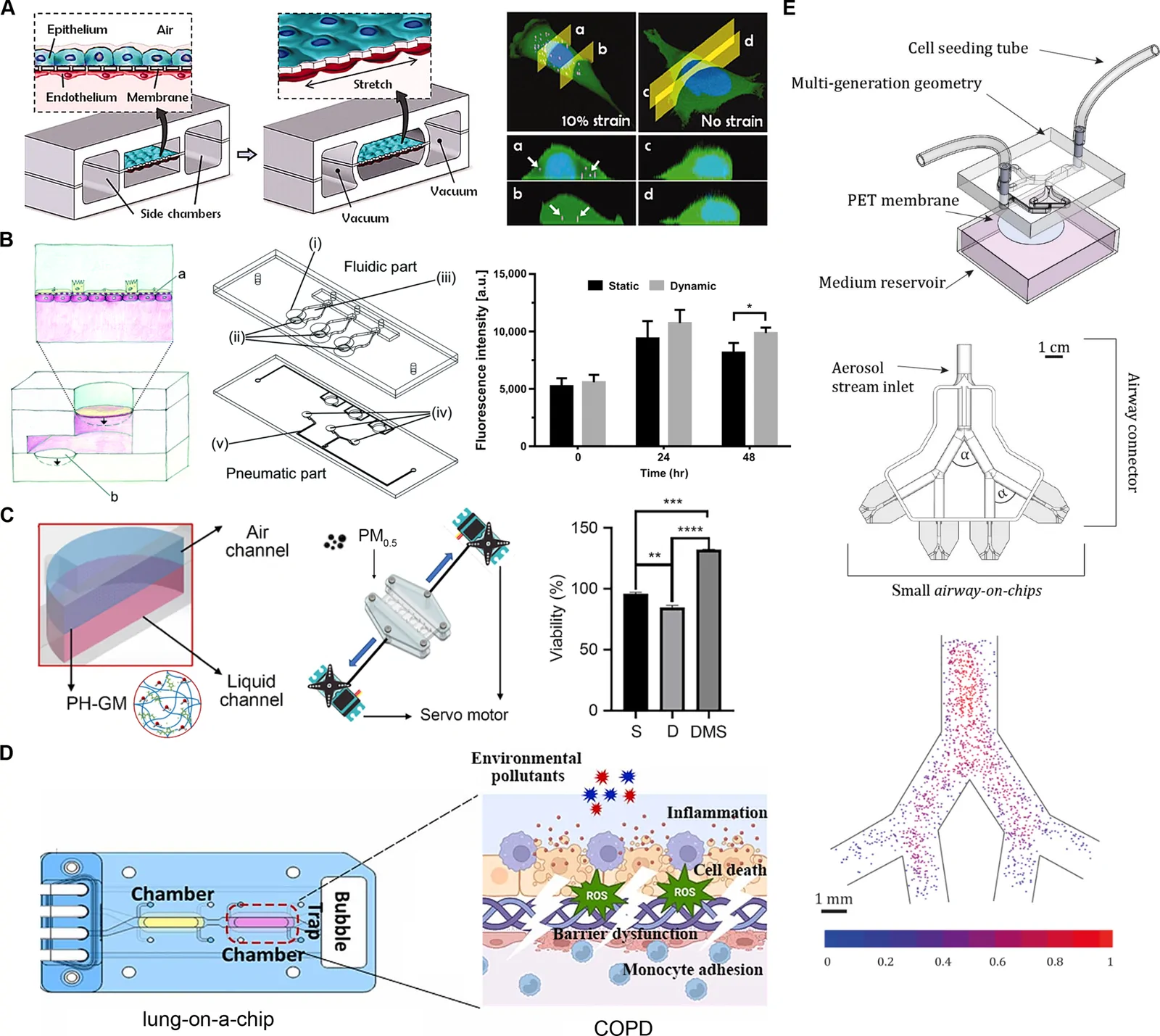
LOCs for investigating PM-induced pulmonary effects. (A) Microfabricated lung mimic device with ECM-coated porous PDMS membrane recapitulated alveolar–capillary barrier and physiological breathing stretch; 10% mechanical strain enhanced cellular uptake of NPs (magenta) vs. static conditions, as illustrated by representative sections (a–d) through fluorescent confocal images (arrows: internalized NPs; green: cytoplasm; blue: nucleus). Adapted from Huh et al. [92]. (B) Alveolar sac-mimicking LOCs with bioartificial alveolar membrane (a) subjected to 3D cyclic mechanical strain via electro-pneumatic micro-diaphragm; (b) cyclic stretching enhanced cellular metabolic activity vs. static controls [(i) alveolar culture wells, (ii) porous flexible membranes, (iii) basolateral chambers, (iv) micro-diaphragms, (v) pneumatic microchannels]. Adapted from Stucki et al. [93]. (C) Lung epithelium-on-a-chip incorporating a PH-GM membrane was developed; a servo motor generated 10% uniaxial cyclic strain (0.2 Hz) to mimic respiratory motion; quantitative analysis demonstrated that the higher effect of PM_0.5_ exposure was observed on cells cultured under DMS conditions compared with those under dynamic and static conditions. Adapted from Kaya and Yesil-Celiktas [94]. (D) LOCs recapitulating alveolar–blood barrier structure/functions with integrated immune cells were built; PS-NP exposure associated with COPD was investigated. Adapted from Yang et al. [96]. (E) Tree-like airway-on-chip with symmetric branching geometry reproduced adult small-airway anatomy and realistic airflow patterns; aerosol deposition was quantified using fluorescence imaging and visualized as normalized concentration heat maps, where warmer colors indicate regions of higher particle accumulation. Adapted from Elias-Kirma et al. [98].

Currently, multiple studies have utilized LOCs to evaluate complex responses following exposure to pulmonary air pollutants [85–88]. Notably, researches have reported that the physiological mechanical signals like the expansion and contraction of alveoli during simulated human respiration can significantly influence the morphology, differentiation, formation of tight junctions, and cytokine secretion of epithelial and endothelial cells [89–91]. In 2010, Huh et al. [92] first microfabricated and validated the LOC model. The chip (a) features 2 parallel microchannels separated by a flexible polydimethylsiloxane (PDMS) membrane coated with ECM proteins, supporting the culture of human alveolar epithelial and pulmonary endothelial cells on its top and bottom surfaces to simulate the alveolar–capillary barrier; and (b) includes 2 hollow side chambers, enabling cyclic vacuum application to stretch the flexible side walls and the membrane with adhered cells, simulating respiratory movements (Fig. 6A). In nanotoxicology research, the team discovered that cyclic mechanical strain enhanced NPs uptake in alveolar epithelial and endothelial cells, exacerbating the lungs’ toxic and inflammatory responses to NPs. It is evident that PM toxicity studies based on static models have intrinsic limitations, potentially leading to a severe underestimation of their actual health risks. In addition, Stucki et al. [93] designed an LOC that simulated the pulmonary parenchymal environment, consisting of a fluidic part and a pneumatic part (Fig. 6B). The chip modeled the human pulmonary alveolar barrier and diaphragm, reproducing the 3D cyclic strain caused by respiratory movements. Their research discovered that cyclic stretching profoundly impacted the permeability of the epithelial cell layer. Furthermore, primary human alveolar epithelial cells cultured in dynamic mode exhibited significantly higher metabolic activity than those cultured in static mode. This confirmed the vital role of respiratory-induced mechanical strain in pulmonary research. In 2024, a research team developed lung epithelium-on-a-chip consisting of PDMS layers along with a thin, flexible, and transparent ionic liquid-based poly(hydroxyethyl) methacrylate gel membrane (PH-GM), applying mechanical stretching with 10% strain and 0.2 Hz frequency to both sides of the chip (Fig. 6C) [94]. The study examined the effects of PM_0.5_ on the chip under static, dynamic, and dynamic mechanical stress (DMS) conditions. Results revealed that the higher effect of PM_0.5_ exposure was observed on cells cultured under DMS conditions compared with those under dynamic and static conditions, highlighting the critical role of cyclic breathing motions in PM toxicity assessment. Furthermore, the maintenance of pulmonary immune homeostasis relies on the coordinated action of various immune cells [42]. Consequently, incorporating the immune system into the LOCs is crucial for investigating the effects of PM on pulmonary diseases [95]. To further explore the association between COPD and polystyrene nano-plastics (PS-NPs), Yang et al. integrated macrophages and circulating THP-1 cells in the LOCs (Fig. 6D) [96]. Research found that as PS-NP concentrations increased, cell viability and TEER levels decreased markedly. PS-NPs traversed the alveolar–capillary barrier into the bloodstream, inducing oxidative stress and inflammatory responses. Concurrently, expression of α1-antitrypsin, a well-established marker of COPD, was significantly decreased, indicating that PS-NP exposure increases the risk of COPD development.

In addition to studying the pathological effects of PM on the lungs, LOCs can also be used to simulate its deposition in the airways under physiological respiratory flow conditions. It is known that the coupling of respiratory flow and lung structure can modulate PM deposition outcomes [97]. A study team proposed a novel airway-on-chip platform to mimic PM deposition within the airway tree structure, finding that LOCs utilizing airflow-driven particle transport better reflected gravity-induced settling behavior of PM at varying flow velocities. The fluorescence imaging quantified particle deposition via color-coded heat maps, where warmer colors indicate regions of higher particle accumulation (Fig. 6E) [98]. The model revealed heterogeneous deposition patterns throughout the airway branches, enabling quantitative assessment of PM transport and regional deposition in distal lung structures. With technological advancements, OOCs are increasingly moving toward integration, miniaturization, and real-time capabilities. The unique architectures of LOCs enable real-time, in situ monitoring of the physiological state of lung tissue on a chip and its response to PM exposure [99]. TEER is the most common type of electrical sensing in LOCs and can be used to quantify the integrity of the air–blood barrier [100]. Jabbar et al. [101] designed a human lung distal airway chip model incorporating TEER sensors and a portable microscope for continuous monitoring. Results demonstrated that PM_10_ exposure significantly elevated levels of pathological biomarkers IL-13, IL-6, and MUC5AC, indicating increased incidence of asthma and COPD within the chip. Furthermore, connecting or integrating the LOCs with other OOCs such as liver-on-a-chip and heart-on-a-chip onto a single platform enables the construction of multi-organ-on-a-chip (MOC) [102,103]. MOC holds promise for further investigating systemic interactions among multiple organs following pulmonary exposure to PM [104], providing mechanistic insights into the systemic health hazards of PM.

Among currently available in vitro models, LOCs offer the highest degree of physiological complexity. Through the integration of microfluidic flow, ALI condition, multicellular coculture, and breathing-like mechanical stimulation, LOCs can address scientific questions that are difficult to investigate using conventional static models. These include the effects of respiratory motion on particle deposition, barrier dysfunction, particle translocation across the air–blood interface, and dynamic inflammatory responses. LOCs enable the evaluation of both molecular and physiological responses. Barrier integrity can be assessed through TEER, permeability assays, and tight junction protein expression. Dynamic endpoints such as particle translocation, immune cell recruitment, endothelial activation, and mechanotransduction-associated signaling pathways are particularly relevant for investigating PM-induced dysfunction under physiologically realistic conditions. The integration of embedded sensors further enables real-time monitoring of metabolic and inflammatory responses.

Although the primary focus of this review is the size-dependent effects of PM on pulmonary toxicity, it is important to note that PM exposure does not occur in isolation in real-world environments. Increasing evidence suggests that PM can synergistically interact with respiratory pathogens, including viruses and bacteria [105]. In this context, advanced in vitro platforms such as organoids and LOCs have emerged as valuable tools to investigate host–environment–pathogen interactions under controlled conditions [12]. These models enable the simultaneous incorporation of environmental particulates and infectious agents, thereby providing a more physiologically relevant framework for studying complex respiratory disease mechanisms.

### A perspective of LOCs

Despite demonstrating tremendous potential, LOC technology still faces considerable challenges toward rapid advancement and large-scale implementation. First is the material bottleneck. While PDMS, the material used in most LOCs, facilitates microfluidic construction, its inherent hydrophobicity limits cell adhesion and exhibits an affinity for hydrophobic small molecules [106]. This may affect the actual exposure concentration and toxic effects of PM. Surface modifications such as oxygen plasma treatment of PDMS offer one solution, though they are not entirely effective. Consequently, alternative biomaterials are frequently incorporated into LOCs [107–109]. The use of synthetic materials, natural materials, or combinations thereof holds promise for overcoming this challenge. Moreover, mechanical forces play a crucial role in lung development, normal function, and pathological changes [90]. A substantial advantage of LOCs over other in vitro models is its ability to achieve dynamic respiratory motion. However, most current breathing simulation models have poor biomimetic properties, primarily achieved by applying alternating vacuum pressure across a biological membrane [92,94]. In vivo, alveolar deformation occurs within highly curved sac-like structures and is characterized by spatially heterogeneous strain distributions, tissue anisotropy, and complex mechanical interactions among alveolar epithelial cells, ECM, and the surrounding capillary network, all of which collectively regulate alveolar biomechanics and tissue homeostasis [10]. In contrast, planar membrane stretching typically produces relatively uniform strain fields that may oversimplify the mechanical microenvironment experienced by alveolar cells. Therefore, the second challenge focuses on simulating the 3D curved expansion of alveoli in vitro. Previous studies have developed porous culture membranes with micro-curved surfaces [110,111], and some researches have integrated vacuum chambers beneath the biofilm layer to drive biofilm movement [93,112], thereby achieving more biomimetic respiratory movements. Nevertheless, current designs often involve complex fabrication processes, limited scalability, reduced experimental throughput, and difficulties in achieving reproducible deformation profiles. Additionally, accurately integrating breathing-associated deformation with vascular perfusion, immune cell recruitment, and realistic aerosol exposure remains technically challenging. Future engineering efforts should focus on developing 3D deformable alveolar structures capable of generating physiologically realistic strain patterns while simultaneously incorporating multicellular interactions, ALI, and real-time sensing technologies. Such advances will be essential for improving the predictive power of LOCs in PM toxicology and respiratory disease research. Last but not least, the current design, manufacturing, and operational procedures for LOCs remain inconsistent. The lack of standardization limits the comparability of data across different research teams.

As discussed above, each in vitro model offers unique advantages and faces specific challenges in simulating PM exposure and its effects on lung health. Moving forward, there is a growing demand for more advanced and integrated in vitro models to better recapitulate the complex interactions between PM and lung tissues, as well as to support clinical PM toxicity assessment and drug screening. Firstly, multi-technology integration will become the primary development direction. Previous reports have proposed combining the controllable directed differentiation of organoids with microfluidic chip technology to construct more biomimetic “next-generation” in vitro models [113,114]. In 2026, Luk et al. [115] constructed a single-donor human induced pluripotent stem cell-derived lung-on-chip. This chip not only reconstructed the multicellular structure of alveolar epithelium–endothelium–macrophages and simulated authentic breathing motion of lung tissue, but also avoided immune and transcriptional background discrepancies caused by heterologous coculture. This demonstrates that synergistically combining organoid and OOC technologies can achieve a “1 + 1 > 2” effect. Secondly, with the rapid advancement of artificial intelligence (AI) technology, research integrating AI with LOCs is gaining momentum [116,117]. Deep learning models demonstrate outstanding performance in image recognition and have been widely applied to interpret microfluidic detection images [118]. George [119] employed machine learning methods to analyze LOC images, achieving automated classification of different cellular states and chip culture quality with markedly higher accuracy than manual assessment. The author’s work illustrates AI’s potential in automated image analysis, quality control, and model standardization. Finally, beyond research utility, LOCs offer important clinical value. They enable human-specific PM toxicity evaluation and high-throughput drug screening for PM-induced lung injury [120]. Technological progress in LOCs will drive personalized toxicity assessment and targeted drug development, supporting precise diagnosis and treatment of PM-related lung injury [121].

**Fig. 7.**
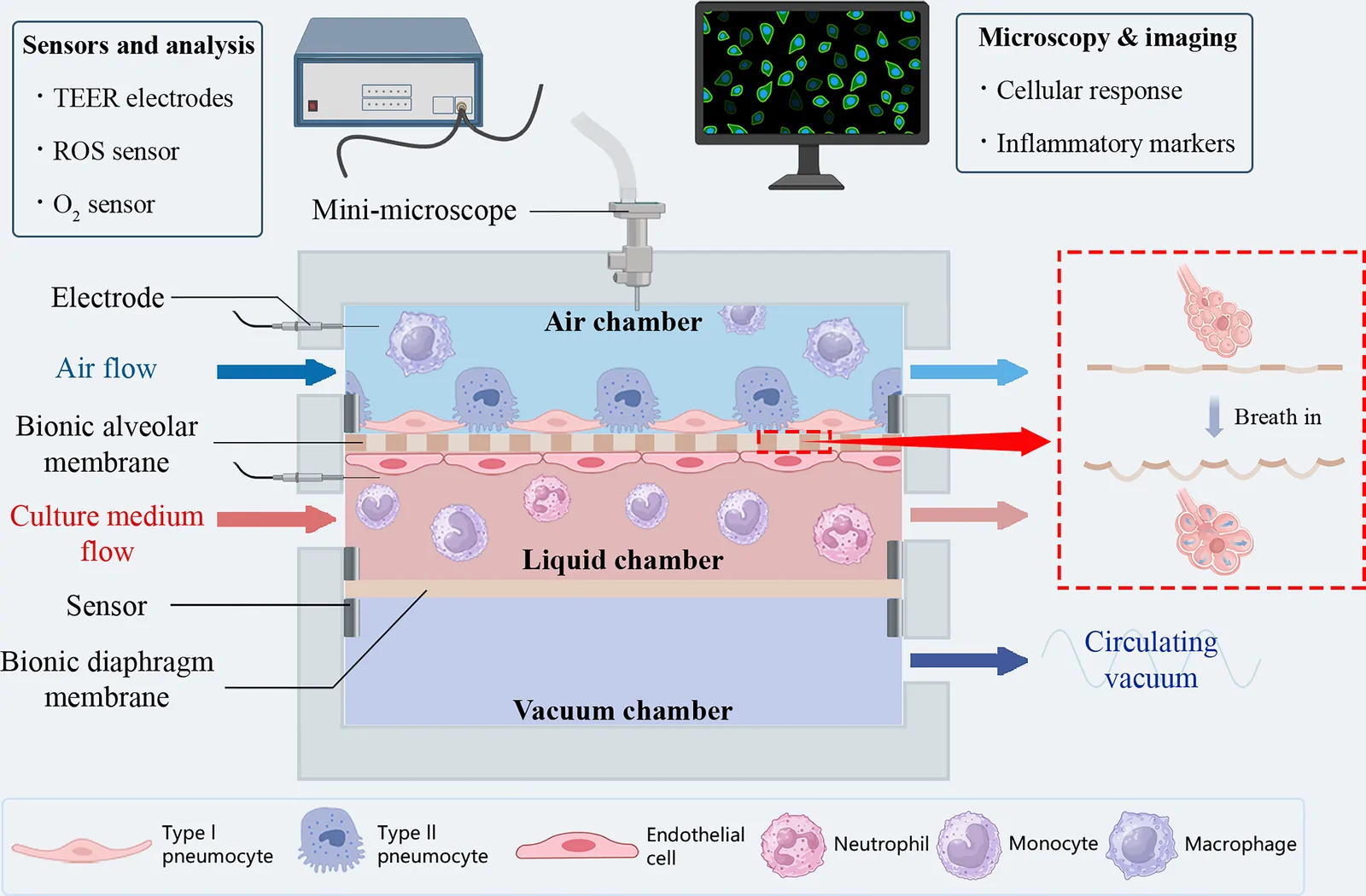
Author-generated schematic diagram illustrating the design of ideal LOCs. Created with BioRender.com.

As such, to enhance the physiological relevance of an LOC model for studying the effects of PM on pulmonary diseases, a 3-chambered structure can be designed. The top chamber serves as a gas pathway for introducing PM-containing aerosols, simulating gas exchange between the lungs and the external environment; the middle chamber is continuously perfused with culture medium via a connected peristaltic pump to reproduce blood flow-related fluid shear stress and maintain cellular nutrient supply; the bottom chamber induces 3D deformation of the flexible bio-membrane through periodic vacuum-driven, thereby simulating physiological respiratory movements. Notably, bio-membrane design is critical for enhancing the model’s biomimetic properties. Its geometry and mechanical properties should reflect authentic alveolar characteristics by constructing a porous culture membrane with micro-curved surfaces. At the same time, surfactant should also be taken into account, as they have a critical impact on particle deposition, interfacial transport, and pulmonary immune responses. Additionally, the LOCs can be integrated with electrodes and sensors to enable real-time monitoring of parameters such as the integrity of the air–blood barrier (measured by TEER), O_2_ levels, and ROS. To observe the transmission or conduction of mechanical signals, pressure-sensing devices can be installed in all 3 chambers. Finally, through microscopic imaging technology, we can observe dynamic changes in cells in real time (Fig. 7). Moreover, with recent advances in AI, this LOC system enables the direct, simultaneous tracking of interrelationships between complex functional parameters, including barrier function, mechanical property changes, immune responses, and cell morphology, during PM exposure and drug screening applications.

Despite marked advances in LOC models, the lack of standardized experimental protocols remains a major obstacle to data reproducibility and cross-study comparison. Future efforts should focus on harmonizing key experimental parameters, including PM exposure metrics (particle number, mass concentration, surface area, and deposited dose), aerosol generation and delivery systems, ALI culture conditions, and breathing-related mechanical stimulation protocols. Standardized functional endpoints are equally important and should include barrier integrity measurements (e.g., TEER and permeability), oxidative stress indicators, inflammatory cytokine profiles, and particle translocation assessments. To facilitate broader adoption and regulatory acceptance, multicenter validation studies using common operating procedures and well-characterized reference particulate materials should be encouraged. Furthermore, the integration of real-time sensing technologies into advanced LOC platforms may enable continuous monitoring of physiological responses while reducing variability associated with endpoint-specific assays. In combination with standardized multi-omics analyses and harmonized data reporting practices, these approaches could establish a more robust framework for evaluating PM-induced pulmonary toxicity. Such standardization efforts are consistent with the highly integrated and intelligent next-generation LOC platform proposed in Fig. 7 and will be essential for improving the predictive power and translational relevance of future PM toxicology research.

## Summary and Outlook

Air pollution has become an important public health issue that urgently needs to be addressed globally, with PM closely linked to the onset and progression of various pulmonary diseases. While animal models still crucial in system-level studies, their species differences and high experimental costs have driven the evolution of in vitro lung models toward greater physiological relevance and translatability. In this review, we summarize the major in vitro models used to study the impact of PM on lung diseases. The reconstruction of lung tissue is challenging due to its complex structure, mechanical properties, and dynamic physiological environment. Early studies predominantly relied on traditional 2D models, which are simple, easy to operate, and reproducible, making them suitable for high-throughput screening and basic toxicity assessments. Nevertheless, these models struggle to replicate the 3D structure, cellular heterogeneity, and the true microenvironment of lung tissue. With the advancement of tissue engineering technologies, 3D bioprinting models have improved cellular arrangement and tissue layering to some extent, but limitations remain in dynamic breathing stimulation and long-term functionality maintenance. Organoid models, relying on the self-organizing capabilities of stem cells, better replicate the cellular composition and certain developmental features of lung tissue, showing higher physiological relevance in disease mechanism studies. However, their lack of key physiological factors such as blood flow and breathing events limits their ability to simulate real exposure conditions. In contrast, LOC models can recreate crucial physiological features of the lungs, such as the air–blood barrier and respiratory stimuli, making them more biomimetic in vitro models. Building upon this foundation, the LOCs still require further optimization in structural design and functional integration. For instance, constructing a multichamber structure could enable synergistic regulation of gas phase exposure, liquid perfusion, and mechanical traction. Concurrently, the biomimetic design of the bio-membrane should incorporate alveolar microstructural features to more authentically replicate the biomechanical environment where PM interacts with cellular and tissue interfaces.

Looking ahead, the development of in vitro models for PM-induced pulmonary toxicology is expected to move toward deep integration of multiple technologies, as well as convergence with emerging approaches such as AI. With the continued advancement of organoids, 3D bioprinting, and OOC technologies, future in vitro models are anticipated to achieve higher levels of structural complexity, physiological relevance, and dynamic functionality. Meanwhile, the integration of multi-omics analyses, high-content imaging, and microfluidic platforms, together with the application of AI and machine-learning algorithms for data integration and pattern recognition, is expected to substantially enhance the efficiency and predictive power of elucidating PM-induced lung injury mechanisms. Such interdisciplinary integration will not only facilitate the construction of lung models that more closely recapitulate the human pulmonary microenvironment but also provide new opportunities for high-throughput toxicity assessment, individualized environmental health risk prediction, and the development of precision intervention strategies.

Beyond improving our understanding of environmental health risks, studies of PM-induced pulmonary toxicity may also provide valuable insights for the development of inhalable nanotherapeutics. Many of the biological processes investigated in PM research, including particle deposition, mucus penetration, surfactant interactions, epithelial uptake, barrier translocation, and immune modulation, are directly relevant to pulmonary drug delivery. In addition, advanced in vitro platforms such as organoids and LOCs offer opportunities to simultaneously evaluate efficacy, safety, and biodistribution of inhaled nanomedicines under physiologically relevant conditions. Consequently, advances in PM toxicology and pulmonary nanomedicine are increasingly interconnected and may mutually accelerate the development of safer and more effective inhalation-based therapies.
